# Depression of Complement Regulatory Factors in Rat and Human Renal Grafts Is Associated with the Progress of Acute T-Cell Mediated Rejection

**DOI:** 10.1371/journal.pone.0148881

**Published:** 2016-02-29

**Authors:** Kazuaki Yamanaka, Yoichi Kakuta, Shuji Miyagawa, Shigeaki Nakazawa, Taigo Kato, Toyofumi Abe, Ryoichi Imamura, Masayoshi Okumi, Akira Maeda, Hiroomi Okuyama, Masashi Mizuno, Norio Nonomura

**Affiliations:** 1 Department of Urology, Osaka University Graduate School of Medicine, Suita, Osaka, Japan; 2 Division of Organ Transplantation, Department of Surgery, Osaka University Graduate School of Medicine, Suita, Osaka, Japan; 3 Department of Urology, Tokyo Women's Medical University, Shinjuku-ku, Tokyo, Japan; 4 Department of Nephrology and Renal Replacement Therapy, Nagoya University Graduate School of Medicine, Nagoya, Aichi, Japan; Universidade de Sao Paulo, BRAZIL

## Abstract

**Background:**

The association of complement with the progression of acute T cell mediated rejection (ATCMR) is not well understood. We investigated the production of complement components and the expression of complement regulatory proteins (Cregs) in acute T-cell mediated rejection using rat and human renal allografts.

**Methods:**

We prepared rat allograft and syngeneic graft models of renal transplantation. The expression of Complement components and Cregs was assessed in the rat grafts using quantitative real-time PCR (qRT-PCR) and immunofluorescent staining. We also administered anti-Crry and anti-CD59 antibodies to the rat allograft model. Further, we assessed the relationship between the expression of membrane cofactor protein (MCP) by immunohistochemical staining in human renal grafts and their clinical course.

**Results:**

qRT-PCR results showed that the expression of Cregs, CD59 and rodent-specific complement regulator complement receptor 1-related gene/protein-y (Crry), was diminished in the rat allograft model especially on day 5 after transplantation in comparison with the syngeneic model. In contrast, the expression of complement components and receptors: C3, C3a receptor, C5a receptor, Factor B, C9, C1q, was increased, but not the expression of C4 and C5, indicating a possible activation of the alternative pathway. When anti-Crry and anti-CD59 mAbs were administered to the allograft, the survival period for each group was shortened. In the human ATCMR cases, the group with higher MCP expression in the grafts showed improved serum creatinine levels after the ATCMR treatment as well as a better 5-year graft survival rate.

**Conclusions:**

We conclude that the expression of Cregs in allografts is connected with ATCMR. Our results suggest that controlling complement activation in renal grafts can be a new strategy for the treatment of ATCMR.

## Introduction

It is known that the classical pathway (CP) of complement participates in antibody mediated rejection (ABMR) and C4d, a metabolite formed by CP activation, is deposited in peritubular capillaries (PTC) over a period of time. Therefore, C4d can be used as a criterion for scoring ABMR [1]. On the other hand, acute T-cell mediated rejection (ATCMR) has been considered to be associated with the activation of complement to a lesser extent. However, some studies reported a significant negative impact on the locally synthesized complement components in grafts in ATCMR. Serinsoz et al. reported an increased C3 expression in both ATCMR and ABMR, and Pratt et al. reported the locally synthesized C3 is eliminated using a C3^-/-^ mouse, resulting in a modulated renal allograft rejection and regulated T cell responses [2, 3]. It has also been reported that a deficiency of and the inhibition of the C5a receptor (C5aR) prolongs renal allograft survival, reduces apoptosis and attenuates the infiltration of inflammatory cells [4, 5].

In the process of complement activation, C3 convertases cleave C3 into C3a and C3b. The production of C3a leads to the interactive activation of antigen presenting cells, T cells and mast cells [6, 7]. Moreover, it drives T cell differentiation, proliferation and expansion [8]. Antagonism of the C3a receptor (C3aR) can also induce functional changes and induce the production of regulatory T cells [9, 10]. On the other hand, the deposition of C3b on the surface of the pathogen is a target for the action of mononuclear phagocytosis. A number of complement regulatory proteins (Cregs) that are anchored on the cell surface manage complement activation. In rodents, such Cregs include complement receptor 1-related gene/protein-y (Crry), decay accelerating factor (DAF: CD55) and CD59. Crry is a special type of Cregs that is produced in rodents and has functions of both a membrane cofactor protein (MCP: CD46) and DAF, but mainly shows MCP function. MCP and DAF act to promote the cleavage of C3b and C4b and to dissociate C3 convertases, respectively, and CD59 to block the formation of a membrane attack complex (MAC) [11, 12]. The causative role of complement has been studied in many kidney diseases, since the kidney controls complement mediated attack by the expression of Cregs on mesangial and tubular cells [13–15]. However, the expression of MCP by renal tubular cells during ATCMR has not been examined in detail. The majority of circulating C3 is synthesized in the liver [16, 17]. However, the renal allograft is a significant source of extrahepatic C3, and C3 secretion is also increased during rejection [18, 19]. The action of local complement components remains to be fully elucidated.

The purpose of this study was to examine the role of complement components produced by renal allografts and the importance of Cregs during ATCMR as related to clinical prognosis.

## Materials and Methods

### Ethics statement

All procedures were performed in accordance with the principles of the Guidelines of Animal Experimentation at Osaka University (approval number J005694-003). Clinical data were collected from the Osaka University Medical Hospital renal transplant and pathology databases. The clinical study was approved by the institutional review board at Osaka University (approval number: 15103).

### Animals

Male Lewis (LEW) (RT1^l^) rats and Dark Agouti (DA) (RT1^avl^) rats, weighing 200 to 250 g, were purchased from Japan SLC Inc. (Shizuoka, Japan) and were maintained under standard conditions until used in experiments. The animals were fed a standard diet and water *ad libitum*. All surgical procedures were performed under anesthesia induced by inhaling Isoflurane (abbvie GK, Japan). We observed rats twice a day during the post-procedure. The Analgesic agent Meroxicam (Wako, Japan) was administered appropriately to manage postoperative pain by subcutaneous injection. Rats were euthanized by inhaling an excessive amount Isoflurane. The procedures for rats were designed and carried out in a manner consistent with the highest scientific, humane, and ethical principles.

### Animal Experimental Protocols

Fully major histocompatibility complex-disparate kidneys from DA rat donors as allogeneic models [number in group (n = 6)] and LEW rats as syngeneic models (n = 3) were transplanted orthotopically into the left side of the nephrectomized LEW recipients. The right native kidney of the recipient was also removed after the transplantation. No immunosuppressive agents were provided. The kidneys and livers were collected from both rat models at each time point (1, 3 and 5 day after transplantation). For the anti-Cregs mouse monoclonal antibodies (mAbs) experiments, the rats were divided into three groups: (i) anti-Crry mAb 0.25 mg/body, (ii) anti-CD59 mAb 0.5 mg/body and (iii) control IgG1 0.5 mg/body. Mouse mAbs against rat Crry (mAb 5I2) and CD59 (6D1) were previously characterized [20–22] as IgG1. Isotype mouse IgG1 was purchased from Bio X cell (West Lebanon, NH) as a control. In the treated groups, each mAb was administered to the graft intra-arterially before the left nephrectomy of the donor. After clamping the suprarenal aorta, the left renal vein was sheared, and a catheter was inserted from the infrarenal aorta to the ostium of the left renal artery. Each graft of DA rat was then perfused with saline. Each Ab was next injected into the graft kidneys. After clamp of the left renal vein, the grafts were transplanted into LEW recipients.

### Selection of clinical cases

All specimens of the renal allograft biopsies were obtained at the Osaka University Medical Hospital between 1989 and 2012 as a retrospective cohort study. None of the transplant donors were from a vulnerable population and all donors or next of kin provided written informed consent that was freely given. The study group consisted of 67 renal transplant biopsies that were originally selected for the diagnosis of ATCMR type I and type II, according to the Banff 1997 and 2007 Classification of Renal Allograft Pathology [1, 23], and sufficient paraffin-embedded tissue was available for the study. The major reason for performing the biopsies was allograft dysfunction. Although some cases required more than one biopsy, only the first biopsy samples for ATCMR were used in this study. The immunosuppression regimen usually involved the use of a calcineurin inhibitor (CNI), mycophenolate mofetil and prednisone. ATCMR is generally treated with glucocorticoids and deoxyspergualin. All the biopsies were taken before antirejection treatment. Graft function was determined by serum creatinine (sCr) measurements, at the time of the biopsy, 2 and 12 months after the treatment. Judged by the level of sCr, a graft that was lost before 12 months after the biopsy was excluded. No cases of death with a functional kidney graft were observed.

### Quantitative Real-Time RT-PCR (qRT-PCR)

Total RNA was extracted from the kidney and the liver using an RNeasy plus Mini Kit (Quiagen, Hilden, Germany), and the isolated total RNA was then reverse transcribed to complementary DNA (cDNA) using the PrimeScript RT reagent Kit (TAKARA Bio Inc., Shiga, Japan), following the manufacturer’s guidelines. The PCR primer sets were used for cDNA amplification of C1q, C4, C3, C3aR, C5, C5aR and CD59 as previously described [24–26], and the primers for complement factor B (FB), C9, Crry and β-actin were purchased by TAKARA Bio Inc. Real-time reverse transcription PCR was performed on a Thermal Cycler Dice Real Time System TP800 (TAKARA Bio Inc.) with SYBR premix Ex Taq II (TAKARA Bio Inc.). Each sample was analyzed in duplicate using the conditions recommended by the manufacturer. Each mRNA level was normalized to the β-actin mRNA level using the comparative ΔΔthreshold cycle method using the manufacturer’s software. The results of qRT-PCR are reported the as relative mRNA expression level compared to that in naïve tissue.

*Complement components are identified according to World Health Organization recommendations (1981) and the reference of Complement nomenclature 2014 [27].

### Histological Evaluation

#### For rat samples

The rat specimens were fixed by immersion in 4% paraformaldehyde and then embedded in paraffin. The tissues were cut into 3-μm thick sections. Each section was stained with hematoxyline and eosin (H&E) and Periodic acid-Schiff (PAS) stain according to standard protocols. The samples obtained from rats were snap-frozen, 3-μm thick sections were prepared with a cryostat and fixed in acetone. The primary staining of tissue was done using an unconjugated antibody and was carried out overnight at 4°C followed by the secondary antibody. The secondary staining was done at room temperature for 15 minutes in the dark. To investigate complement deposition, the frozen sections were incubated with polyclonal rabbit anti-rat C4d Ab (1:50, Hycult Biotech, Uden, The Netherlands) and the polyclonal rabbit anti-rat FB Ab (1:200, Novus Biologicals, Colorado, USA), followed by incubation with Alexa Fluor 488-labeled goat anti-rabbit IgG (H+L) (1:500, Invitrogen, California, USA). For the detection of complement regulatory proteins, frozen sections were incubated with the mouse anti-rat Crry mAb as described above, and the mouse anti-rat CD59 mAb (1:50, Acris Antibodies GmbH, Herford, Germany) followed by incubation with Alexa Flour 488-labeled goat anti-mouse IgG (1:500, Invitrogen). We used the goat anti-rat TIM-1 Ab (1:50, R&D System Inc., Minnesota, USA) as a marker of proximal tubules in identifying the localization of CD59 in renal tubules, followed by incubation with Alexa Fluor 488-labeled Donkey anti-mouse IgG H&L and Alexa Fluor 594-labeled Donkey anti-rabbit IgG H&L (1:500, both of which were obtained from Abcam, Cambridge, UK). In addition, cells in the samples were double-stained with DAPI (Sigma-Aldrich, Missouri, USA). Controls using only the secondary antibodies were all included in the immunofluorescence assays (S1 Fig). We evaluated the immunochemistry findings except for these background possible non-specific interfering compounds. Micrographs of stained sections were acquired on a fluorescence microscope (Keyence BZ-9000 fluorescence microscope, Keyence, Osaka, Japan) with BZ-II analyzer software (version 1.2.0.1, Keyence). A semi-quantitative scale was used to score the deposition of FB, Crry and CD59 in each sample.

#### For clinical samples

To evaluate the expression of complement factors and Cregs in the kidneys, 10–20 Fields were randomly observed under a fluorescence microscope. The human biopsy specimens were fixed by immersion in a neutral 10% (w/v) formalin buffer solution and then embedded in paraffin. The tissues that were embedded in paraffin were cut into 3-μm thick sections. Each section was stained with H&E, PAS, Periodic acid-methenamine-silver and Elastic-Masson stain according to standard protocols. Immunohistochemical staining was performed using the LSAB+System-HRP (Dako, Glostrup, Denmark), according to the manufacturer’s instructions. The mouse anti-human MCP mAb (mAb M177) was kindly provided by Prof. Seya Tsukasa (Hokkaido Univ, Japan). Staining scores were classified semi-quantitatively as follows: 0, tissue specimen with little or very low staining and/or less than 10% of the renal tubules; 1 (weak), 10~50%; 2 (moderate), more than 50%; 3 (strong). The low MCP expression group involved score 0 and 1 specimens, and the high MCP expression group involved score 2 and 3 specimens. The number of MCP positive cells was counted, in at least 5 randomly selected grafts (Magnification; ×200).

#### Statistical analysis

For statistical analyses, the JMP 10.0.2 (SAS institute Inc., North Carolina, USA) and GraphPad Prism 5 (Graphpad Software, California, USA) software programs were used. Data are expressed as the mean [standard error of the mean (SEM)]. We used Levene’s test to assess the equality of variances between the two groups. When the results of Levene's test were statistically significant, we assumed that the variances of the two groups were equal, and the equal-variances t-test was then used to assess the differences between two groups. On the other hand, when not significant, we assumed the values to be unequal, and then used the un*e*qual-variances t-test. The expression of complement factors and receptors in the days after the allograft transplant was evaluated by Welch’s test followed by Tukey’s honestly significant difference test (Tukey’s HSD test). Data are expressed as the median (minimum-maximum) in clinical groups, and the Mann–Whitney nonparametric test was used to determine a significant difference between groups and binary variables were analyzed by the χ^2^ test or the Fisher exact test. Graft survival was evaluated by means of a Kaplan-Meier survival analysis, and the differences in survival were measured by the log-rank test. A p-value of less than 0.05 was considered to be significant.

## Results

### Animal model

#### Changes in local complement production and activation of the alternative pathway in allografts

**Fig 1:**
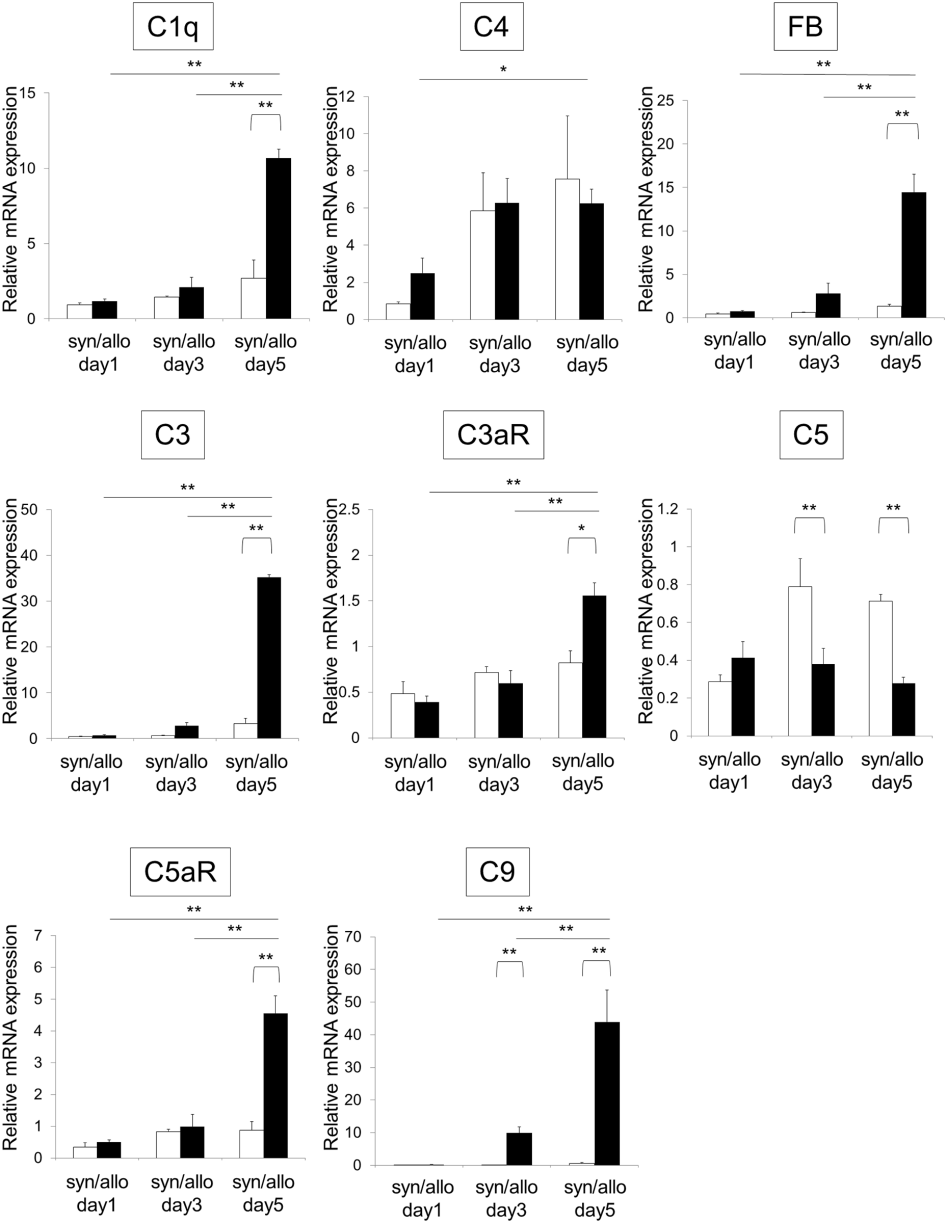
Quantitative real-time PCR analysis reveals an up-regulated expression of complement components in the allogeneic graft model. White bars and black bars show syngeneic grafts (syn) and allogeneic grafts (allo), respectively. Data are shown as the mean (SEM). *p < 0.05 and **p < 0.01 compared with the corresponding value for the syngeneic graft models and the mRNA levels of complement proteins and receptors in the days following allograft transplant. Statistical significance was assessed by the t-test and Tukey’s HSD test. Relative expressions of C1q, FB, C3, C3aR, C5aR and C9 mRNA in the allografts were significantly elevated, compared to syngeneic grafts at day 5 after transplantation. For all analyses, n = 6 for allografts and n = 3 for isografts.

The mRNA expression of each complement component was compared between syngeneic and allogeneic grafts (Fig 1). The mRNA levels of the C1q and FB in allografts were significantly elevated at day 5, compared with syngeneic grafts. However, the C4 mRNA levels were raised during the days following the transplant in a similar way for both groups, thus not presenting any difference between them. In addition, a significant increase in the mRNA levels of C3, C3aR and C5aR at day 5 was observed in the allografts. Concerning C9, the levels were significantly elevated both at day 3 and 5. On the other hand, the C5 mRNA levels in the allografts remained constant in the days 1, 3 and 5 after the transplant. This behavior was different from the observed in the syngeneic group, which had the C5 mRNA expression elevated in the days 3 and 5. Most of the mRNA levels of each complement component were dynamically elevated in the allograft, but not in the recipient liver (S2 Fig).

**Fig 2:**
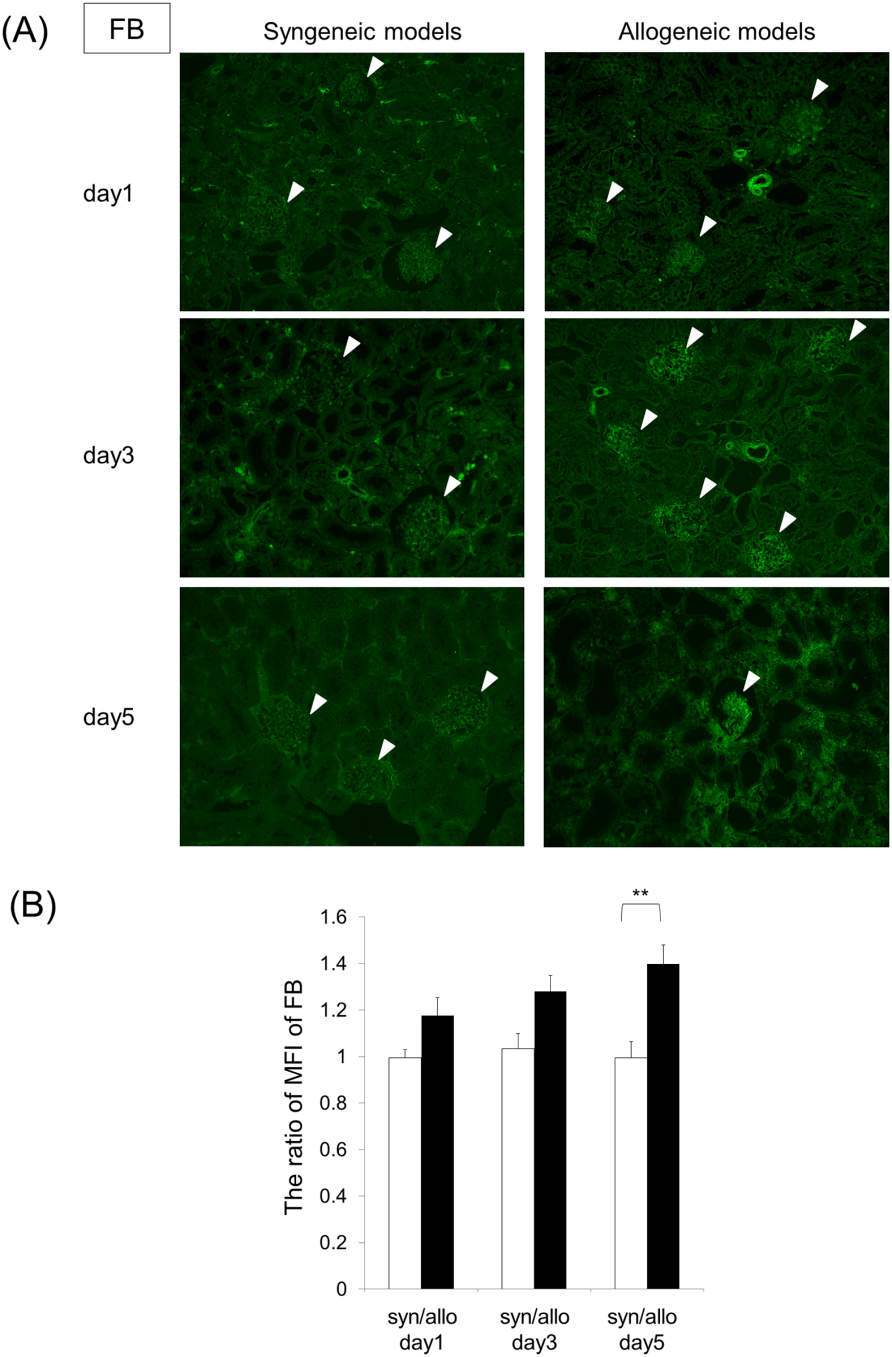
Deposition of complement FB in the allograft models. (A) Allograft groups showed higher levels of FB deposition in glomeruli when compared to the syngeneic ones. There was no FB deposition in the tubules. FB deposition was analysed by fluorescence immunochemical staining. (Magnification, X200). White arrow heads indicate FB-positive glomeruli. (B) Mean fluorescence intensity (MFI) of FB = MFI of glomeruli / MFI of renal tubules per field × MFI of glomeruli / MFI of renal tubules per field. The ratio of MFI of FB = MFI of syngeneic or allogeneic model / MFI of naïve kidney. White bars and black bars show syngeneic grafts and allogeneic grafts. Data are shown as the mean (SEM). **p < 0.01 compared with the corresponding value of the syngeneic graft models. Statistical significance was assessed by the t-test and Tukey’s HSD test.

Fluorescence immunehistochemical findings showed that C4d was not deposited in the PTC (data not shown). The immunofluorescence shows us that FB is deposited in the grafts, suggesting a possible activation of the alternative pathway (Fig 2A). The mean fluorescence intensity (MFI) of FB in the allogeneic models was significantly higher than in the syngeneic models at day 5 after transplant (p = 0.0092) (Fig 2B). The MFI of FB in the days following the allograft transplant was not significantly increased (p = 0.2592).

#### The expression of Cregs is decreased in the allograft model

**Fig 3:**
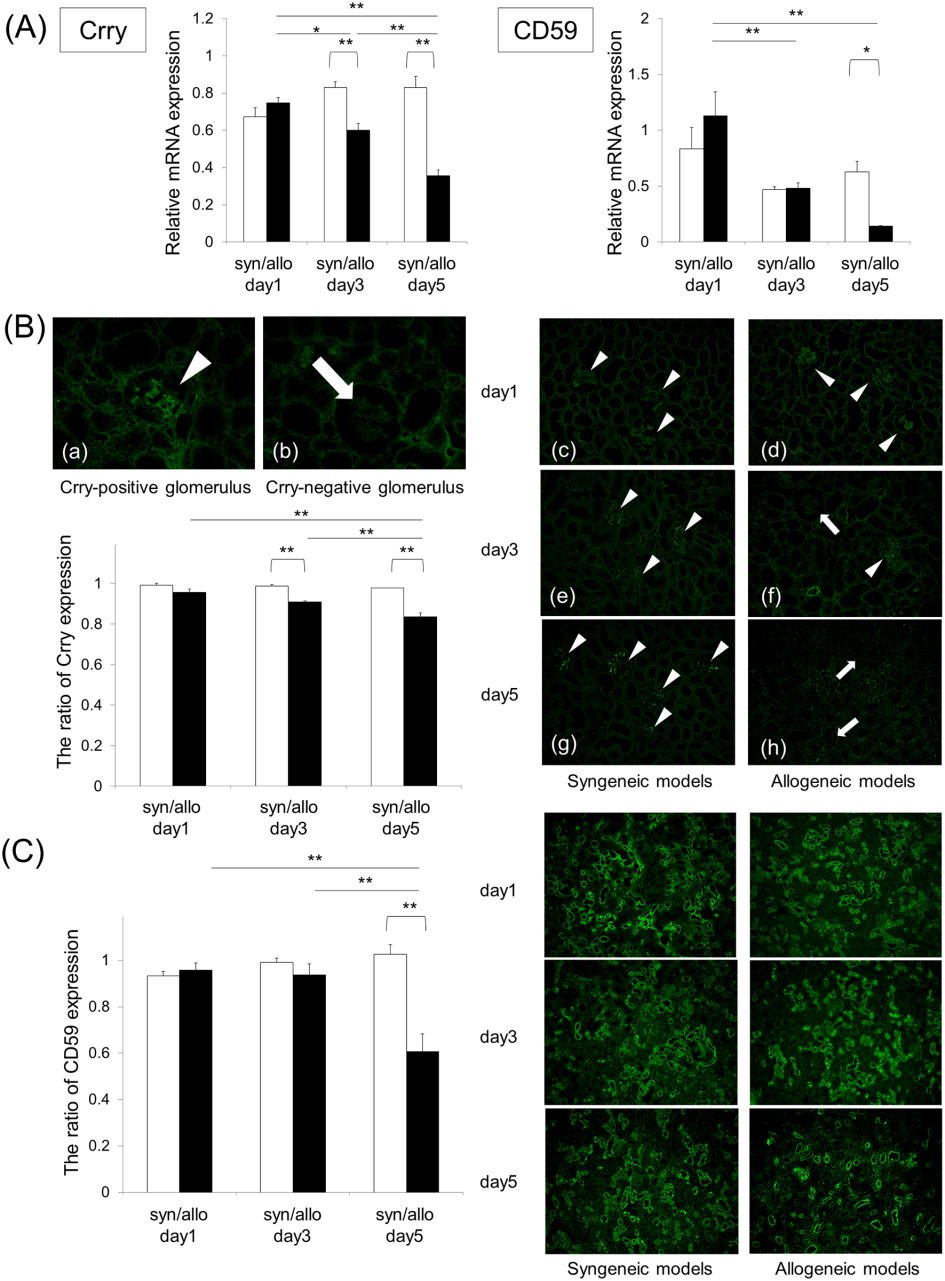
The expression of Cregs is diminished in the allograft model with the progression of ATCMR. White bars and black bars show syngeneic grafts and allogeneic grafts, respectively. Data were shown as the mean (SEM). *p < 0.05 and **p < 0.01 compared with the corresponding value of the syngeneic graft models and the levels of Cregs following the days of allograft transplant. Statistical significance was assessed by the t-test and Tukey’s HSD test. (A) qRT-PCR showed significantly diminished expression levels of Crry and CD59 mRNA in the allografts, compared to syngeneic grafts, at day 5 after transplantation. (B) The arrow head and the arrow indicate Crry-positive and Crry-negative glomeruli [Magnification: (a) and (b) X400, (c) ~ (h) X200]. Crry was not expressed in renal tubules. The ratio of Crry expression = number of Crry-positive glomeruli / total number of glomeruli per sample. The expression of Crry in the glomeruli of allograft groups was significantly decreased at day 3 and 5 after transplantation in fluorescence immunochemical staining, compared to syngeneic groups. (C) CD59 was expressed in the glomeruli and proximal tubules. The expression of CD59 = the area of CD59-positive glomeruli and renal tubules / total area of glomeruli and renal tubules per field. The ratio of CD59 expression = CD59 expression of syngeneic or allogeneic models / CD59 expression of naïve kidney. (Magnification, X100). CD59 expression in glomeruli and tubules of the allografts significantly declined on posttransplant day 5.

In contrast to the increased expressions of complement components of the AP and terminal pathway, the expressions of mRNA of Crry and CD59 in the allografts were significantly lower than those in the syngeneic grafts at various points (Fig 3A). On immunofluorescence staining, the expressions of Crry in the glomeruli and CD59 in the glomeruli and proximal tubules were maintained in the syngeneic models (Fig 3B and 3C and S3 Fig). However, in allogeneic models the expressions of Crry were significantly decreased at day 3 and 5, and CD59 was also decreased at day 5.

#### The survival period is shortened when anti-Cregs mAbs are administered

**Fig 4:**
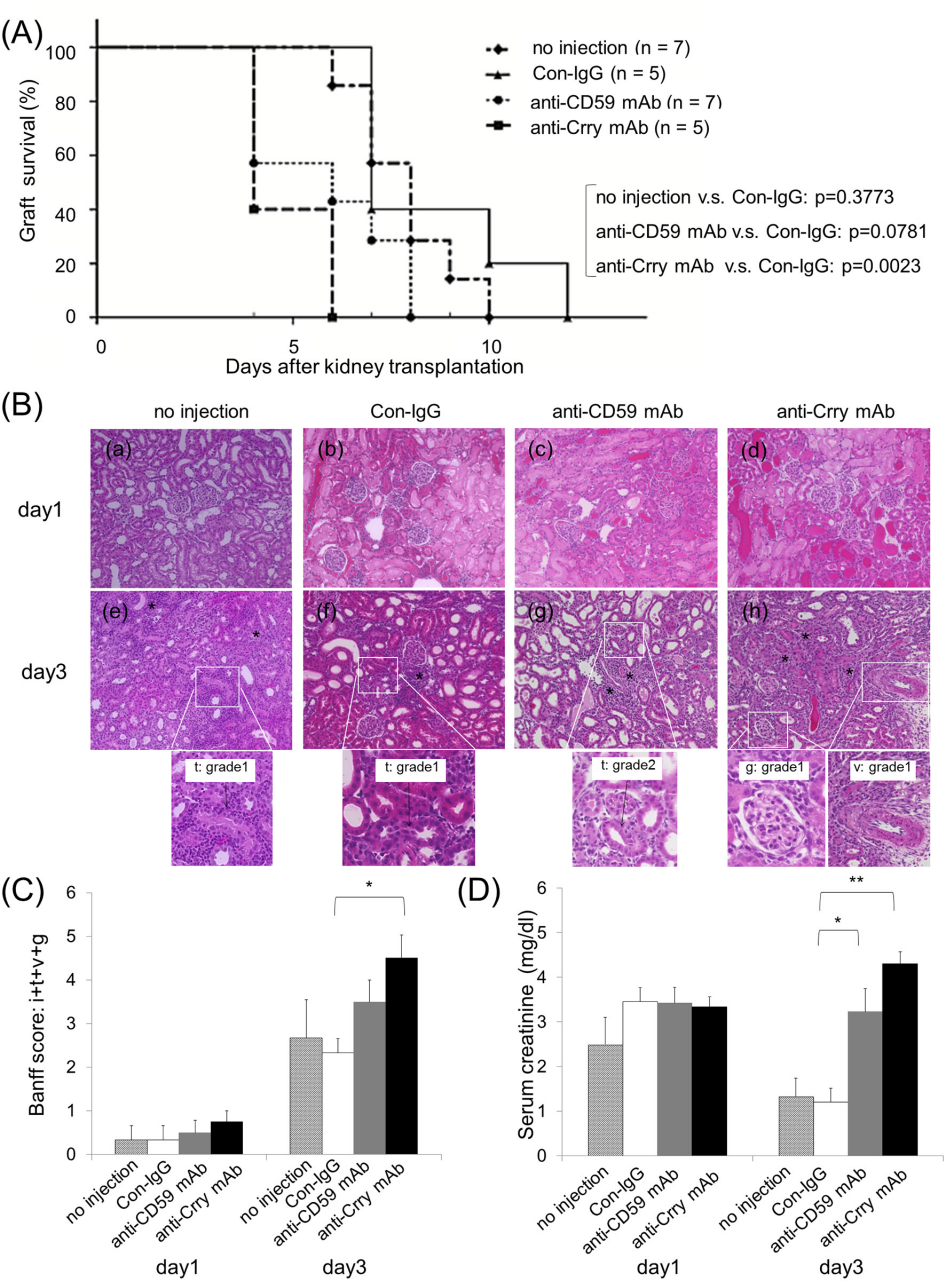
Inhibition by anti-Crry and anti-CD59 mAbs shortens allograft survival. When the grafts were harvested from DA donors, anti-Cregs mAb were administrated to the grafts. The grafts were then transplanted to LEW recipients. (A) The graft survival time was observed in all groups; the allogenic control group administrated no drug (n = 7), anti-CD59 mAb group (n = 7), anti-Crry mAb group (n = 5) and Control-IgG group (Con-IgG, n = 5). The differences in survival were measured by the log-rank test. (B) Paraffin-embedded sections at day 1 and 3 from each group were stained with H&E to assess the rejection grade (Magnification, X200). [interstitial inflammation (i): *, tubulitis (t), intimal arteritis (v) and glomerulitis (g)] (C) The parameters (i, t, v and g) of Banff criteria relevant to ATCMR was assessed on the H&E staining. The parameters of Banff criteria relevant to a naïve kidney were all 0, indicating normal findings (data not shown). No significant difference was found between the group with control IgG and the group without injection in the parameters (day1: p = 1.000 and day3: p = 0.5993). (no injection group: n = 3, control IgG group: n = 3, anti-CD59 mAb group: n = 4, and anti-Crry mAb group: n = 4). (D) The serum creatinine levels in all groups were determined at the day1 and day3 after transplantation. No significant difference in serum creatinine levels was found between the group with control IgG and the group that was not injected (day1: p = 0.1566 and day3: p = 0.8045). (C, D) Data are shown as the mean (SEM). *p < 0.05 and **p < 0.01 compared with the corresponding value for the control-IgG group. Statistical significance was assessed by the t-test.

To examine the effects on ATCMR on inhibiting Cregs, the recipients were administered the anti-Crry mAb and the anti-CD59 mAb (Fig 4A). No significant difference in graft survival was found between the group with control IgG and the group without any injection [control IgG: 8.60 (1.03) days and no injection: 7.86 (0.51) days, p = 0.3773]. Compared to the IgG control group, the group that was administered the anti-Crry mAb had a significantly shortened median survival period from 8.60 (1.03) days to 4.80 (0.49) days (p = 0.0023). Delivering anti-CD59 mAb also reduced the survival duration to 5.86 (0.70) days, but this value did not reach the level of significance (p = 0.0781).

From histopathological findings, all of the models showed evidence of only acute tubular necrosis without ATCMR at day 1 after transplantation (Fig 4B). On day 3, the control-IgG group revealed a borderline change in Banff criteria, but the anti-CD59 and anti-Crry groups showed relatively elevated findings for ATCMR. Grafts in anti-Crry mAb group were accompanied by vasculitis. On day 3, the anti-Crry mAb group showed significantly higher parameter scores for Banff criteria, but no difference in the scores was found for the anti-CD59 group compared to the control-IgG group (anti-Crry: p = 0.0446 and anti-CD59: p = 0.1345) (Fig 4C). Regarding renal graft function on day 1 after transplantation, sCr levels were elevated in all groups (Fig 4D). While on day 3, the levels were clearly reduced in control-IgG group, the other groups that had received antibodies maintained significantly high levels [anti-CD59 mAb: 3.23 (0.52) mg/dl, anti-Crry mAb: 4.30 (0.27) mg/dl and control-IgG 1.20 (0.32) mg/dl, p = 0.0101 vs.CD59 and p = 0.0002 vs. Crry]. In the light of the above data, it can be concluded that the inhibition of Cregs resulted in a progressively worse ATCMR score, and an impaired graft function.

### Clinical study

#### Patient background

**Fig 5:**
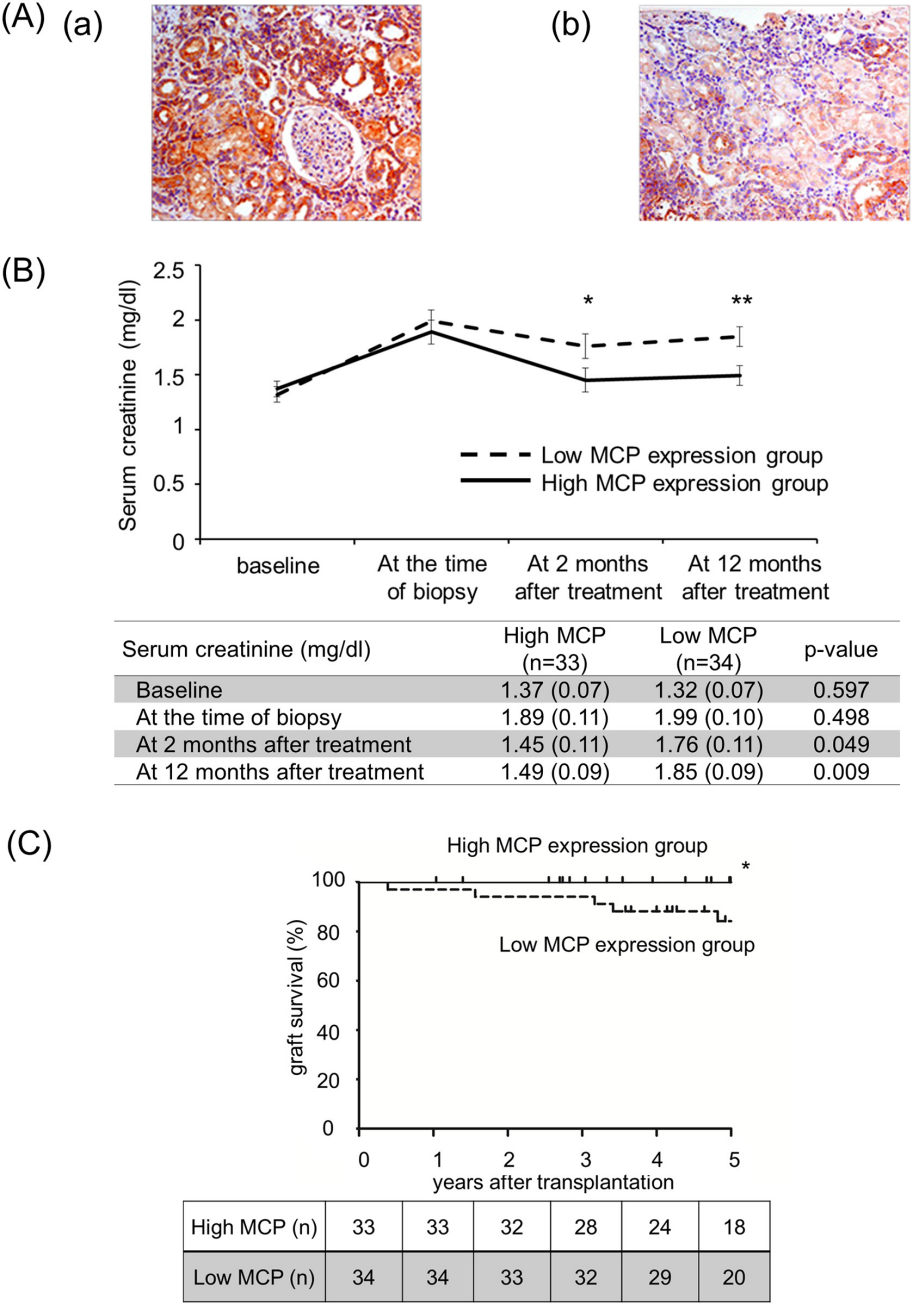
The group with higher MCP expression in the grafts improved graft function. (A) Immunohistochemical stain with mAb to MCP in human renal biopsies with ATCMR. The representative samples were indicated MCP expression on the renal tubuli: high MCP expression (a) and low MCP expression (b) (Magnification, X200). (B) Serum creatinine features of both the high and low MCP expression groups. The treatment effect for ATCMR was estimated by checking the serum creatinine levels. The normal range of serum creatinine levels in males is 0.6–1.2 mg/dl and in females is 0.4–0.9 mg/dl. Data are shown as mean (SEM). *p < 0.05 and **p < 0.01 compared with the corresponding value of Low MCP expression group. Statistical significance was assessed by the t-test. (C) Kaplan-Meier estimates of 5-year graft survival in patients with ATCMR based on the level of MCP expression at the time of biopsy. There is a significant difference in graft survival between the high and low MCP groups (log-rank test; p = 0.0353). *p < 0.05 compared with the corresponding value of Low MCP expression group. The number of patients at risk in each point is shown in the table.

Based on the rat experimental data, a clinical study was next carried out. Demographics and clinical data for 67 patients with indication biopsies are shown in Table 1. The high MCP expression group involved 33 cases, and the low MCP expression group involved 34 cases (Fig 5A). There were no significant differences in the patient backgrounds between the high and low MCP groups (Table 1).

**Table 1:** Patient characteristics. The statistical significances for recipient age, donor age and HLA mismatch was assessed by the Mann–Whitney nonparametric test. The binary variables of the others were analyzed by the χ^2^ test or the Fisher exact test. The data are shown as the median values (minimum-maximum). No significant differences were found in the demographic and clinical data for the transplant patients. HLA: human leukocyte antigen

	High MCP group (n = 33)	Low MCP group (n = 34)	p-value
Recipient sex (male / female)	17 / 16	15 / 19	0.628
Recipient age	45 (19–68)	42.5 (19–69)	0.910
Donor sex (male / female)	12 / 21	13 / 21	1.000
Donor age	52 (17–75)	58 (40–73)	0.830
Donor source (living / cadaveric)	3 / 30	6 / 28	0.476
ABO compatible / incompatible	30 / 3	30 / 4	1.000
Calcineurin inhibitor (CSA / TAC)	15 / 18	21 / 13	0.224
ATCMR (Grade I / II)	25 / 8	24 / 10	0.784
HLA mismatch	3 (0–5)	3 (0–6)	0.738

### Graft function

No difference in sCr levels were found between the patients with the high and low MCP levels of expression before and at the time of biopsy (Fig 5B). After the anti-rejection treatment, patients with a high MCP expression maintained significantly lower sCr levels compared to patients with low MCP expression. The results of a Log-rank test of 5-year graft survival in patients with ATCMR are shown in Fig 5C. Graft survival was significantly improved in patients with high MCP expression compared to those with low MCP expression, 100% vs. 79.4% respectively (p = 0.0353).

## Discussion

The complement system has been recognized as a potential mediator and diagnostic indicator of rejection in kidney transplants [28, 29]. That is, complement components and Cregs appear to play key roles in the immune response, not only during humoral, but also in cellular rejection [30–32]. As a well-known instance, in a mouse renal transplantation model, recipients that received C3-/- kidneys had a long-term graft survival [30]. Our results in the rat experiments also support this view, because ATCMR is strongly related to the local production of complement components. While the majority of the complement components circulating in the blood are produced by the liver [33], significant amounts are also produced by renal allografts, especially during rejection [34].

An ischemic reperfusion injury (IRI) induces the activation of complement via the AP [35–37]. Therefore, because in the animal model of renal transplantation, it is impossible to avoid the effects of IRI, we used allogeneic and syngeneic grafts model which are both equally affected by IRI. Local C3 expression is upregulated in the cellular rejection of renal allografts [2, 3]. The results showed that there was no difference in C4 mRNA expression between the syngeneic and allograft groups. However, both the qRT-PCR and immunofluorescence analysis showed an increased expression and deposition of FB in the allogeneic model in the 5th day after the transplant, which suggests possible AP activation. The expression of both C3aR & C5aR was also elevated. These upregulations, C3 and FB are known to be regulated by inflammatory cytokines, such as IFN-γ, IL-1α, TNF-α or IL-2 [31, 32, 38–40]. Therefore, the expression of complement components and receptors: C3, C3aR, C5aR, FB, C9, C1q, was increased, but not the C4 and C5 expression. This behavior may be related to inflammatory cytokines that are produced during the rejection process [41–43]. However, restrictions apply to our study, since we failed to check which cell in the graft produced complement components. Complement production such as C3 and C6 from inflammatory cells affects cascade activation as well as that from glomerular and tubular cells [19, 44–47]. In addition, concerning the local production of C5 by the grafts, the changes were relatively difficult to understand. The mRNA data was then reexamined by several other pairs of primers for rat C5, but the results were similar. In the rat allografts, C5 expression was not elevated in the days following the transplant. However, the expression of C5aR was upregulated, which indicates that this receptor might possibly be activated by non-locally produced C5a. C5a as well as C3a strongly acts as anaphylatoxin, and exerts inflammatory and chemotactic actions. C5a produces a local injury through contact with C5aR that is expressed on cells in the graft. C5a stimulates T cells and APC, enhances T cell proliferation and diminishes T cell-related apoptosis [8, 48]. Antagonism of C5aR can also induce functional changes and induce regulatory T cells [9, 10]. C5a signaling is prominently involved in accelerating ATCMR. Therefore, C5 should be accorded more interest by researchers and further studied, using other combinations of rats, such as knock-in rats with a marked C5, might be needed to solve this discrepancy.

To prevent tissue injury by unintended complement activation, host membrane Cregs, such as MCP, DAF and CD59 in human, are involved [49–51]. MCP serves as a cofactor for factor I, which then cleaves C4b & C3b that are attached to the cell membrane by the activation of the complement cascade. As a typical case to avoid humoral rejection, recent xenotransplantations using transgenic pigs bearing human MCP showed a prolonged graft survival [52]. Our results using an animal model indicate that the decrease in CD59 and Crry expression in the grafts may be correlated with a possible activation of the complement cascade in the tissue. Although there are no reports showing where CD59 is expressed in renal tubules, our research revealed that CD59 is expressed in the proximal tubules, as evidenced by double staining with CD59 and TIM-1 (S3 Fig). We also assessed the mRNA levels of CD55 (data not shown). The mRNA levels of CD55 in allografts were significantly elevated at day 1 and 5, compared with syngeneic grafts. We performed immunofluorescence staining for CD55 using a polyclonal rabbit anti-rat CD55 Ab. We confirmed that CD55 was expressed at the podocytes in glomeruli and on the surface of erythrocytes, as reported previously [53, 54]. It is also known that a deficiency of CD55 leads to the development of paroxysmal nocturnal hemoglobinuria. CD55 expression was much weaker at podocytes than on erythrocytes. The podocytes are localized to a small region and are observed to be inhomogeneously-distributed. Therefore, it is difficult to correctly evaluate the extent of CD55 expression at podocytes because of the strong influence of those on erythrocytes.

As expected, sCr levels were elevated in all rat allograft groups on day 1 by the effect of IRI in Fig 4D [55]. Subsequently, while the sCr levels in the Control-IgG group fell to a lower level by recovery from IRI on day 3 and had a negligible effect on rejection, the sCr levels in both anti-Cregs mAbs groups maintained a high titer because of progressing rejection even if recovered from IRI.

In general, these results of the animal study are in agreement. Clinical results support the view that kidney grafts with a lower expression of MCP after ATCMR may have a lower cytoprotective capacity for complement activation, and the graft then responds poorly to the treatment. On the other hand, a similar result to ours reported that survival of an allograft with no evidence of ABMR was significantly better in the human case of the diffuse PTC CD55 staining group than in the negative PTC CD55 staining group [56]. Therefore, over all, better graft survival requires the maintenance of an appropriate expression of Cregs at levels sufficient to avoid both ABMR & ATCMR.

A study of complement regulation by common immunosuppressive agents, suggests that they have no influence on local complement synthesis [31, 57]. Therefore, the regulation of complement activity can be considered to be a potential target for the treatment of ATCMR. Although it has not been reported that complement regulatory drugs and recombinant Cregs, including MCP, were used for ATCMR, they are expected to have a potential effect on ATCMR to improve graft outcomes, by regulating complement activation in the local phase [58–60].

## Supporting Information

S1 Fig Controls using only the secondary antibodies without the primary antibodies were performed in the immunofluorescence assays. The tissues stained were naïve kidneys. We evaluated all samples under the same conditions. (a) Alexa Flour 488-labeled goat anti-mouse IgG (1:500), (b) Alexa Fluor 488-labeled goat anti-rabbit IgG (H+L) (1:500), (c) Alexa Fluor 488-labeled Donkey anti-mouse IgG H&L (1:500) and (d) Alexa Fluor 594-labeled Donkey anti-rabbit IgG H&L (1:500) (Magnification, X200).
(TIF)

S2 Fig qRT-PCR analysis in the liver of the syngeneic and allogeneic models. White bars and black bars show complement mRNA of the liver in the syngeneic and allogeneic models, respectively. Data are shown as the mean (SEM). *p < 0.05 and **p < 0.01 compared with the corresponding value of the syngeneic graft models. Statistical significance was assessed by the t-test and Tukey’s HSD test. Complement mRNA expression of liver didn’t exhibit a specific pattern. For the above analysis, we used n = 6 for allografts and n = 3 for isografts.
(TIF)

S3 Fig CD59 expression in the proximal tubules and glomeruli. LEW naïve kidney was stained with CD59 (green), TIM-1 (red) and DAPI (blue). TIM-1 was used as a marker of proximal tubules. (Magnification, X200).
(TIF)
