# Supplementary figures and images for: Depression of Complement Regulatory Factors in Rat and Human Renal Grafts Is Associated with the Progress of Acute T-Cell Mediated Rejection
Source: PLoS One. 2016 Feb 29;11(2):e0148881. doi: 10.1371/journal.pone.0148881 (PMC4771804; doi:10.1371/journal.pone.0148881)

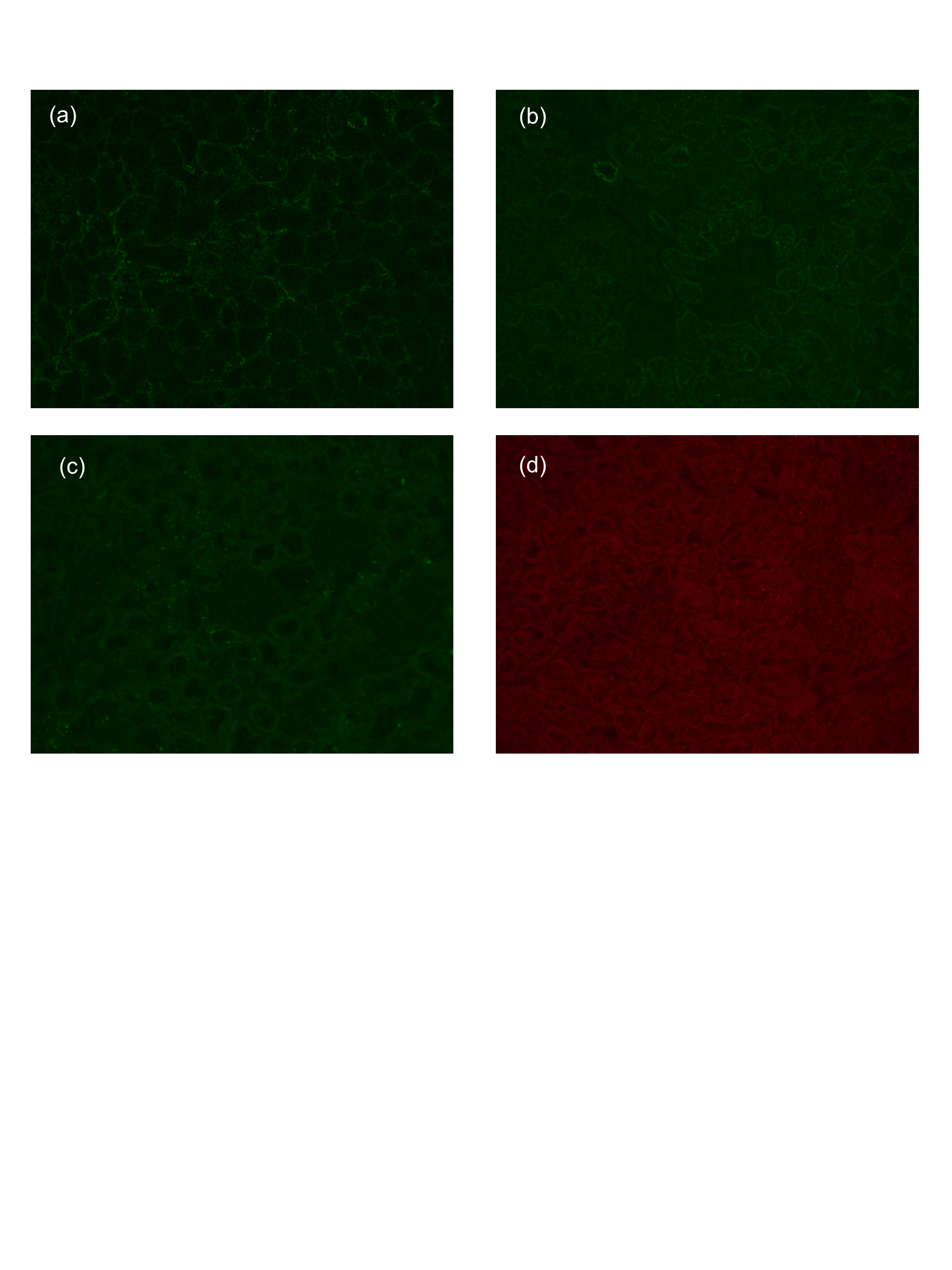

Supplement: S1 Fig — The tissues stained were naïve kidneys. We evaluated all samples under the same conditions. (a) Alexa Flour 488-labeled goat anti-mouse IgG (1:500), (b) Alexa Fluor 488-labeled goat anti-rabbit IgG (H+L) (1:500), (c) Alexa Fluor 488-labeled Donkey anti-mouse IgG H&L (1:500) and (d) Alexa Fluor 594-labeled Donkey anti-rabbit IgG H&L (1:500) (Magnification, X200). (TIF) [file pone.0148881.s001.tif]

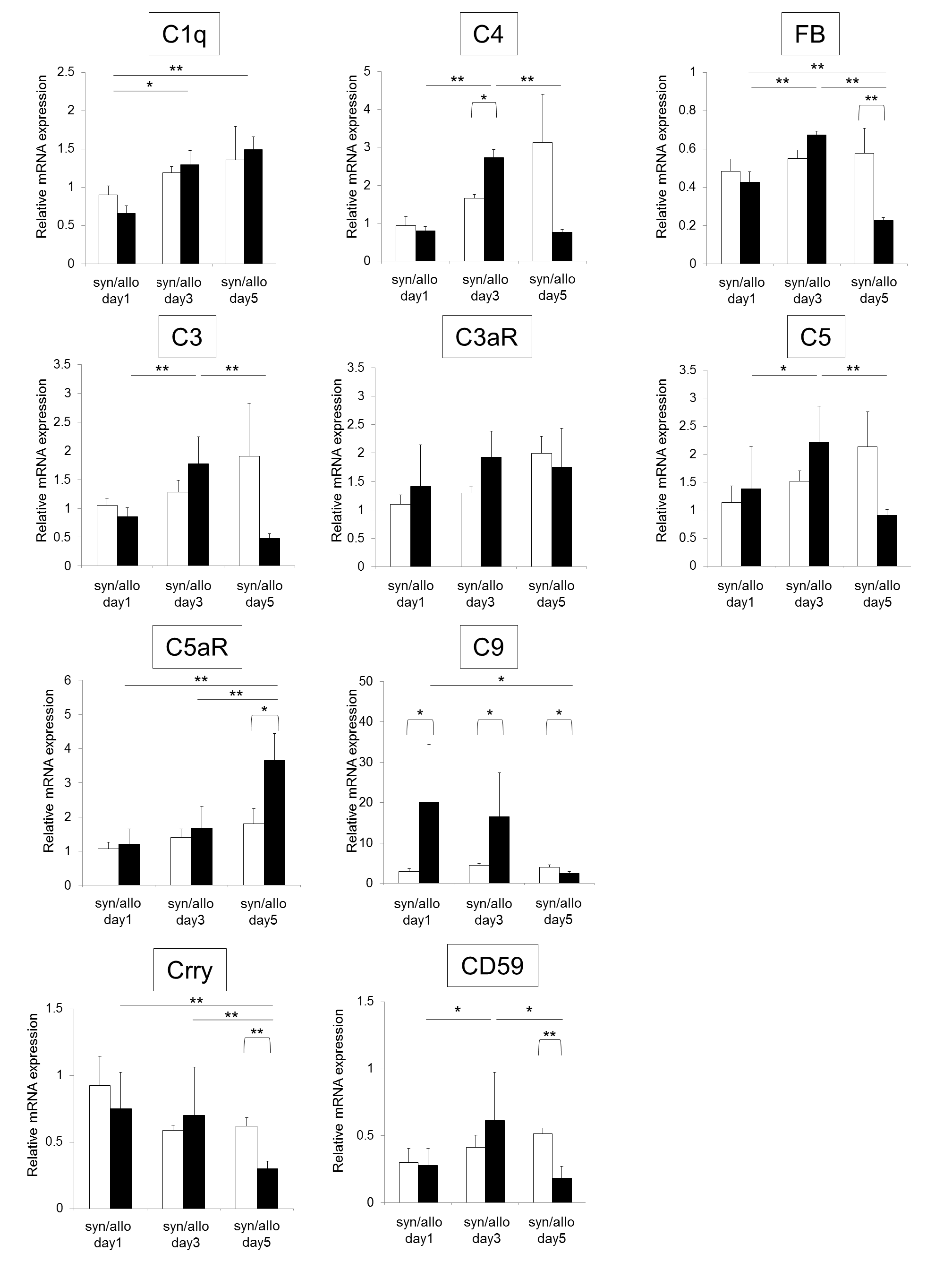

Supplement: S2 Fig — White bars and black bars show complement mRNA of the liver in the syngeneic and allogeneic models, respectively. Data are shown as the mean (SEM). *p < 0.05 and **p < 0.01 compared with the corresponding value of the syngeneic graft models. Statistical significance was assessed by the t-test and Tukey’s HSD test. Complement mRNA expression of liver didn’t exhibit a specific pattern. For the above analysis, we used n = 6 for allografts and n = 3 for isografts. (TIF) [file pone.0148881.s002.tif]

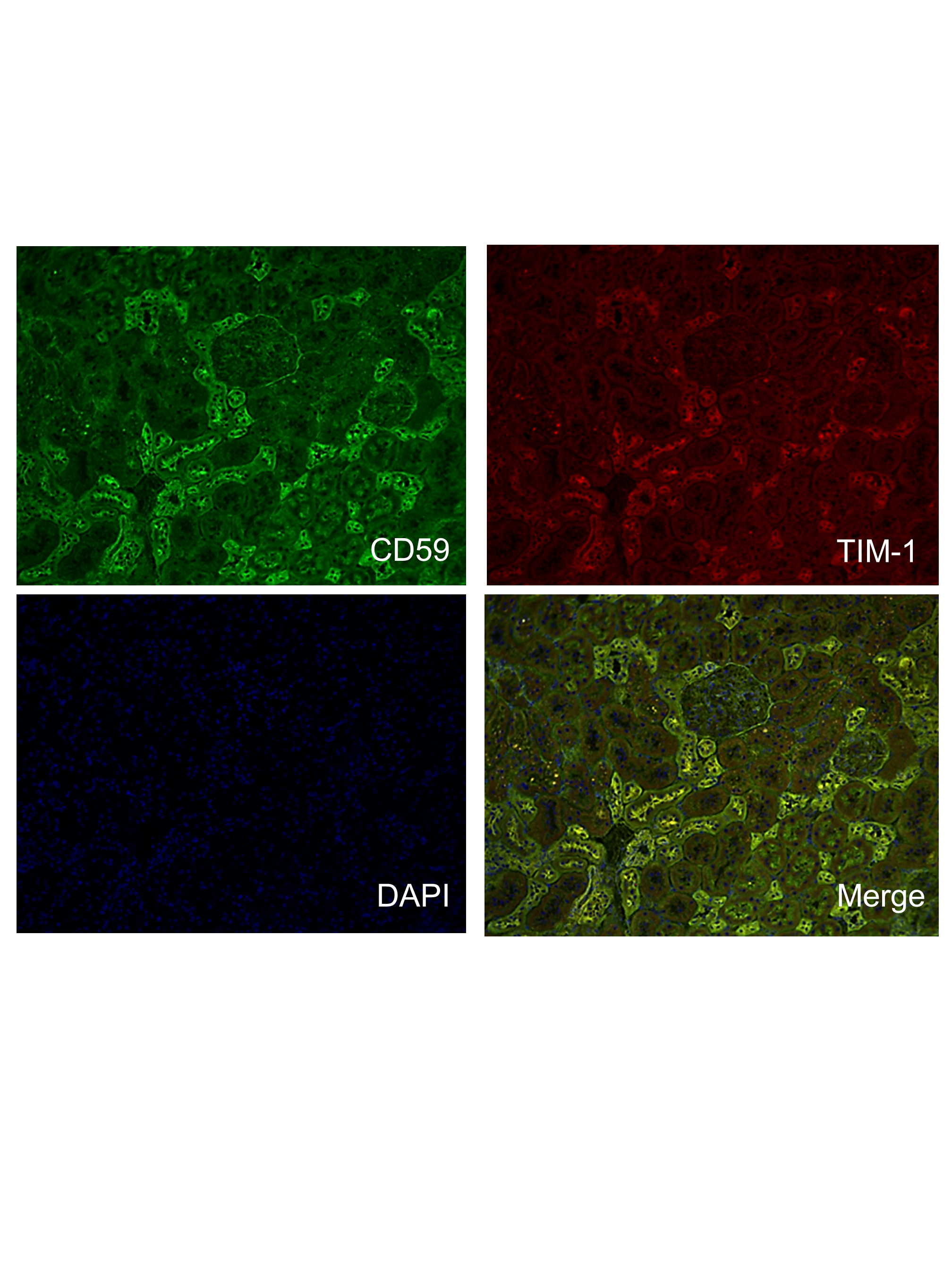

Supplement: S3 Fig — LEW naïve kidney was stained with CD59 (green), TIM-1 (red) and DAPI (blue). TIM-1 was used as a marker of proximal tubules. (Magnification, X200). (TIF) [file pone.0148881.s003.tif]
